# Dynamic Performance and Power Optimization with Heterogeneous Processing-in-Memory for AI Applications on Edge Devices

**DOI:** 10.3390/mi15101222

**Published:** 2024-09-30

**Authors:** Sangmin Jeon, Kangju Lee, Kyeongwon Lee, Woojoo Lee

**Affiliations:** Department of Intelligent Semiconductor Engineering, Chung-Ang University, 84, Heukseok-ro, Dongjak-gu, Seoul 06974, Republic of Korea; jademin96@cau.ac.kr (S.J.); agl0312@cau.ac.kr (K.L.); since69se@cau.ac.kr (K.L.)

**Keywords:** process-in-memory (PIM), emerging non-volatile memory (NVM), performance optimization, data allocating algorithm, low-power design, heterogeneous architecture

## Abstract

The rapid advancement of artificial intelligence (AI) technology, combined with the widespread proliferation of Internet of Things (IoT) devices, has significantly expanded the scope of AI applications, from data centers to edge devices. Running AI applications on edge devices requires a careful balance between data processing performance and energy efficiency. This challenge becomes even more critical when the computational load of applications dynamically changes over time, making it difficult to maintain optimal performance and energy efficiency simultaneously. To address these challenges, we propose a novel processing-in-memory (PIM) technology that dynamically optimizes performance and power consumption in response to real-time workload variations in AI applications. Our proposed solution consists of a new PIM architecture and an operational algorithm designed to maximize its effectiveness. The PIM architecture follows a well-established structure known for effectively handling data-centric tasks in AI applications. However, unlike conventional designs, it features a heterogeneous configuration of high-performance PIM (HP-PIM) modules and low-power PIM (LP-PIM) modules. This enables the system to dynamically adjust data processing based on varying computational load, optimizing energy efficiency according to the application’s workload demands. In addition, we present a data placement optimization algorithm to fully leverage the potential of the heterogeneous PIM architecture. This algorithm predicts changes in application workloads and optimally allocates data to the HP-PIM and LP-PIM modules, improving energy efficiency. To validate and evaluate the proposed technology, we implemented the PIM architecture and developed an embedded processor that integrates this architecture. We performed FPGA prototyping of the processor, and functional verification was successfully completed. Experimental results from running applications with varying workload demands on the prototype PIM processor demonstrate that the proposed technology achieves up to 29.54% energy savings.

## 1. Introduction

With the advent of the artificial intelligence (AI) era, AI technology has made remarkable progress and is being widely applied in real-life scenarios. Particularly, the integration with IoT devices has rapidly expanded the scope of AI technology to edge devices, which are closely connected to the end-user environment. This shift has increased the necessity to process data-centric tasks in AI applications more energy-efficiently and swiftly across various edge devices. Traditional centralized data processing methods, such as cloud computing, have enabled large-scale data processing and storage, but also have several limitations, such as energy consumption due to data transmission, latency, and data privacy concerns [1,2]. To overcome these limitations, computing has gained attention. Edge computing processes data directly on the edge device, that is, close to the data source, reducing network load effectively and minimizing latency caused by data transmission [3].

Since edge devices have more stringent energy constraints compared to centralized data processing methods, energy-efficient data processing is a major challenge [4,5]. In response, various technological efforts have been made to enhance the energy efficiency of edge computing, one of which is the integration of processing-in-memory (PIM) architecture with edge devices [6,7]. PIM performs data processing inside or near the memory array, reducing latency due to data movement and addressing the key design goal of high energy efficiency in edge devices for memory-centric tasks such as AI applications [8]. In traditional computing architectures, data movement between the processor and memory incurs significant latency and energy consumption, but PIM has the potential to alleviate the memory bottleneck by fully utilizing memory bandwidth [9]. However, most current research on PIM has focused on the development of PIM architecture and its integration with existing processors, with performance improvements being evaluated under fixed computational conditions [10,11]. When considering practical applications, there is greater potential for energy efficiency improvements by reflecting dynamic scenarios, where the computational load fluctuates in real time during the runtime of an application. For instance, in autonomous vehicles equipped with convolutional neural network (CNN) applications for object recognition and road condition assessment, the inference workload per hour can vary significantly depending on factors such as weather, traffic, and the movement of surrounding vehicles [12,13]. Using fixed computational resources to meet the maximum performance requirements for all time intervals without accounting for these fluctuations can lead to inefficient energy consumption. Therefore, to maximize the energy efficiency of the PIM architecture in such realistic scenarios, a flexible approach that accommodates the variability in the computational workload is needed, but this area has yet to be deeply explored.

In this paper, we propose a novel PIM architecture that can flexibly respond to real-time variations in the computational workload of edge applications, as well as an operational algorithm to optimize the energy efficiency of the proposed PIM architecture. Firstly, the proposed PIM architecture consists of *PIM modules*, where each PIM module is a fundamental unit of computation, comprising memory and a processing element (*PE*). We introduce two types of PIM modules in the proposed architecture: the low-power PIM (LP-PIM) modules and the high-performance PIM (HP-PIM) modules. In other words, the proposed PIM architecture is a heterogeneous architecture composed of both LP-PIM and HP-PIM modules, providing the capability to flexibly respond to varying computational loads in real time. Next, we propose a data placement optimization algorithm that maximizes the potential of the heterogeneous PIM architecture. This algorithm predicts the changing computational workload of the running application and optimally allocates data to the HP-PIM and LP-PIM modules, thereby improving the energy efficiency of the proposed heterogeneous PIM system. For instance, when the computational workload of the application is low, the system allocates a higher proportion of data to the LP-PIM modules to reduce the workload on the HP-PIM modules, minimizing the dynamic energy consumed by the HP-PIM modules. Conversely, when the computational workload is high, the system actively utilizes the HP-PIM modules to increase the processing throughput of the heterogeneous PIM system. Furthermore, we developed the proposed algorithm by taking into account the time and energy overhead caused by data movement between PIM modules, ensuring that the system meets the computational latency requirements of the application while maximizing energy efficiency.

To verify the functionality and evaluate the effectiveness of the proposed technology, we performed the modeling of the memory device and PE, followed by the register transfer level (RTL) design of the entire PIM processor, including the proposed heterogeneous PIM architecture. Additionally, we conducted experiments using field-programmable gate array (FPGA) prototyping with various testbench scenarios to validate the energy-saving effects of the proposed PIM architecture and data placement algorithm. The results demonstrated that the proposed approach maximizes energy efficiency while meeting the computational latency requirements of applications in edge computing environments. More precisely, the developed PIM processor showed superior adaptability to real-time variations in computational load compared to the baseline PIM architecture-based processor, demonstrating an average energy efficiency improvement of up to 29.54% and at least 21.07%. These results demonstrate the potential of the heterogeneous PIM architecture in edge computing environments and prove that the proposed technology is well suited to maximize the efficiency of edge processors performing AI applications.

The remainder of this paper is organized as follows. In Section 2, we describe the proposed heterogeneous PIM architecture and the computational mechanism of the hardware in detail. Section 3 provides the data placement algorithm for optimizing the energy efficiency of the heterogeneous PIM architecture. Section 4 is dedicated to the experimental work. In this section, we describe the FPGA prototyping of the PIM processor equipped with the proposed PIM architecture, and demonstrate the superiority of the proposed technology by running various scenarios on the developed PIM processor prototype and measuring the results. Finally, the Conclusions summarizes the research findings and the significance of this study.

## 2. Proposed Heterogeneous PIM Architecture for Edge Processors

The PIM architecture is designed to fully utilize memory bandwidth, which can significantly improve the performance of memory-centric applications by alleviating memory bottlenecks. However, when applying such PIM architectures to the processors of edge devices, where power efficiency and battery life are critical, energy efficiency must be carefully considered. While various energy optimization techniques for edge processors have been studied, traditional low-power circuit and design methods determine power efficiency at design time, making them effective only in scenarios where the workload is constant or changes in a periodic and predictable pattern [14,15,16,17]. Moreover, power capping techniques such as workload scheduling or dynamic voltage and frequency scaling (DVFS), which dynamically adjust the power efficiency of processors, introduce additional overhead due to operating circuits and real-time power monitoring [18,19,20]. Therefore, we propose a heterogeneous PIM architecture that can dynamically maximize energy efficiency, even in situations where workloads fluctuate irregularly over time, while delivering high performance in memory-centric tasks such as AI applications.

**Figure 1:**
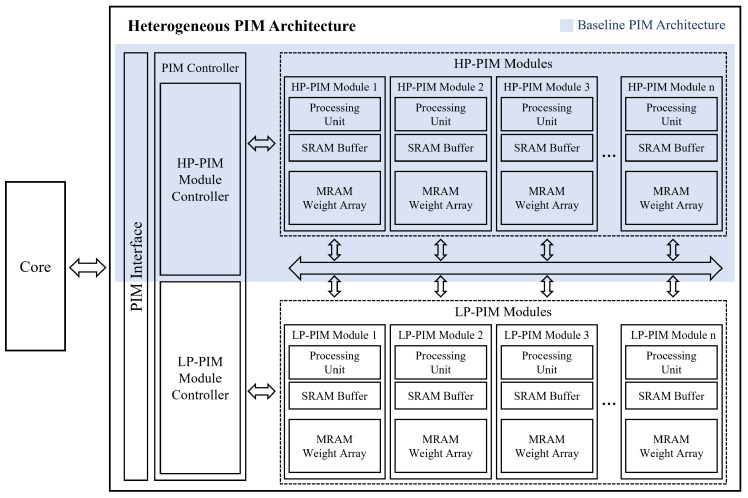
Proposed heterogeneous PIM architecture.

Figure 1 shows the proposed heterogeneous PIM architecture. The gray-colored section in the figure represents the baseline PIM architecture. The overall configuration of the functional blocks in this baseline adopts the basic structure of several previously studied PIM architectures [21,22,23], including multiple PIM modules composed of PEs and memory banks, a controller to manage these modules, and an interface for external communication. The most significant feature of the proposed PIM architecture is the inclusion of two types of PIM modules: HP-PIM, which operates at high performance with high power consumption; and LP-PIM, which operates at low power with low performance. The hallmark of the proposed PIM architecture lies in its integration of two distinct types of PIM modules: the HP-PIM, optimized for intensive computations with higher power consumption; and the LP-PIM, designed to operate at lower power with reduced performance. This configuration allows the heterogeneous PIM to dynamically balance power efficiency and performance. The PIM controller enables each PIM module to independently perform data I/O or computations based on commands received from the core through the interface. Two PIM controllers independently manage the HP-PIM and LP-PIM modules, ensuring stable PIM operation by synchronizing between the PIM modules. The PIM interface between the system and the heterogeneous PIM is designed based on a 32-bit-width AXI interface, facilitating communication with the core. Specifically, the PIM interface either receives PIM operation requests from the core and forwards them to the PIM controller, or notifies the core when PIM operations are completed. This PIM interface is designed as a single channel with a single data path, operating at a data rate of 1.6 Gbps under a 50 MHz system clock frequency.

Meanwhile, the heterogeneous PIM architecture incorporates two types of memory, SRAM and STT-MRAM, each included in the configuration of the PIM modules at the bank level. SRAM primarily serves as a buffer for data to be processed or for the results of computations performed by the PE. Its fast read and write speeds ensure that the PE can quickly access the data required for computation, preventing a decrease in processing speed due to memory access latency. Due to its relatively large footprint, however, SRAM is not suitable for storing large amounts of data, such as the weights of neural networks in AI applications. On the other hand, neural network weights, once trained, are used for inference without further updates until additional training is required. This characteristic makes non-volatile memory (NVM), which retains data even when power is off, an ideal choice for storing such weights [24]. STT-MRAM, in particular, stands out as an NVM with read and write speeds fast enough to be used as cache memory, while consuming less power than DRAM, which requires periodic refreshes. This makes STT-MRAM highly suitable for edge devices [25]. Consequently, we adopted both SRAM and STT-MRAM in the proposed PIM architecture, ensuring that data are stored in the appropriate memory type based on their characteristics.

Next, in designing the heterogeneous PIM architecture, we devised a data storage method and processing flow to minimize data movement overhead. Conventional PIM architectures typically configure independent computation units at the subarray or bank level, whereas in the proposed heterogeneous PIM architecture, the computation unit is the PIM module. The PE within a PIM module thus cannot directly access data stored in another PIM module without the aid of a controller. If data are stored randomly, performance degradation due to data movement overhead becomes inevitable. Since the optimal data storage location varies depending on the types of computations involved in the application, developers must carefully consider this to minimize data movement overhead. To illustrate this, we use computations from convolutional neural network (CNN)-based AI inference models, which are frequently employed in various applications, as an example to explain the data storage method and processing flow in the proposed heterogeneous PIM architecture that minimizes data movement overhead.

**Figure 2:**
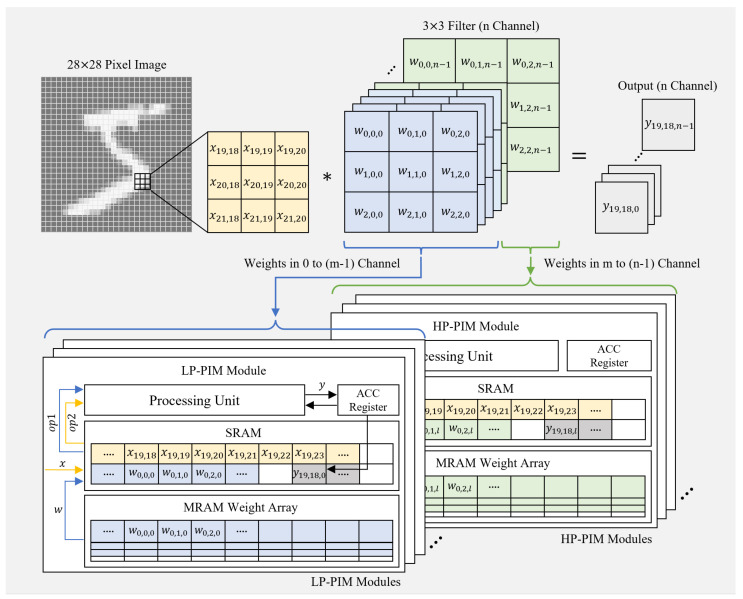
Weight allocation scheme for convolution layers in the proposed heterogeneous PIM architecture.

Figure 2 shows the weight allocation scheme of the heterogeneous PIM for the convolution layer in a CNN. In the convolution layer, the weight (*w*) corresponds to the filter. In the example, where a 28×28-pixel image is used as the input *x*, and *n* output channels *y* are generated through *n* different 3×3 filters, the output for the (i,j) pixel of the input image can be expressed by the following convolution operation:

(1) $$y_{i,\;j,\;k}=\sum_{a=0}^{2}\sum_{b=0}^{2}x_{i+a,\;j+b}\cdot w_{a,\;b,\;k}\;\left(\mathrm{for}\;k=0,1,\cdots ,n-1\right).$$

In this convolution operation, the results of computations between the input data *x* and each filter are independent of one another. To reduce data movement overhead, as shown in Figure 2, the weights can be distributed across the PIM modules on a per-channel basis. Accordingly, the *n* filters are divided between the HP-PIM and LP-PIM modules in a ratio of m:(n−m), with each module storing a portion of the weights. Unlike the distributed storage of weights across the PIM modules, the input data *x* for the convolution layer are broadcast to all PIM modules to allow parallel processing, and are stored identically in each module’s SRAM buffer. During the computation, each PIM module moves the required weights to its SRAM buffer and sequentially feeds the input data and weights to the PE for multiply–accumulate (MAC) operations. The ACC register is used to store intermediate results from the MAC operations, and once the computation for each filter is completed, the output *y* is stored in the SRAM buffer.

**Figure 3:**
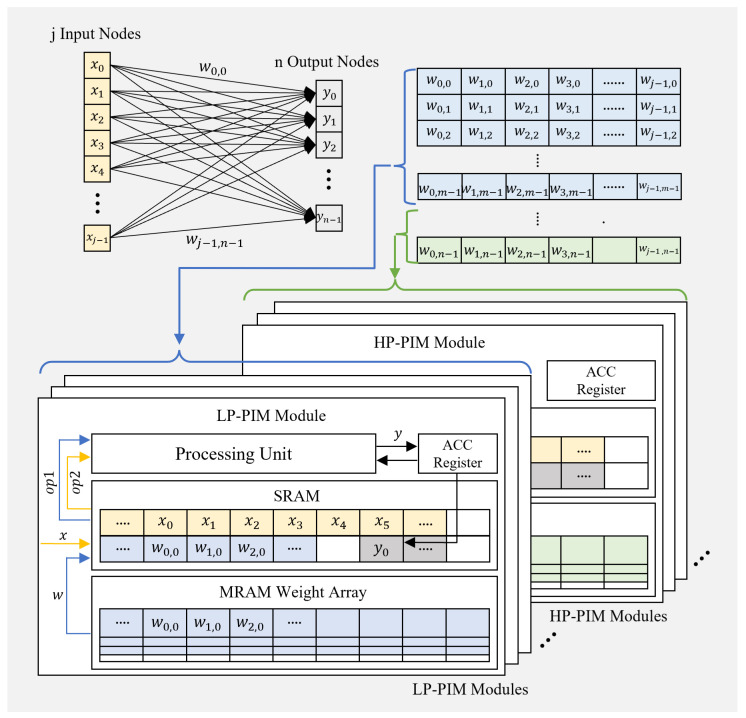
Weight allocation scheme for fully connected layers in the proposed PIM architecture.

Now, turning our attention to the fully connected layer of a CNN, Figure 3 presents the weight allocation scheme for the heterogeneous PIM architecture. In the fully connected layer, the operation involves a matrix–vector multiplication between the input vector *X* with *j* input nodes and the weight matrix *W* of size j×n, producing an output vector *Y* with *n* output nodes. Denoting the elements of *X*, *Y*, and *W* as *x*, *y*, and *w*, respectively, the matrix–vector multiplication at the element level can be described as follows:

(2) $$y_{k}=\sum_{i=0}^{j-1}x_{i}\cdot w_{i,\;k}\;\left(\mathrm{for}\;k=0,1,\cdots ,n-1\right).$$

In the fully connected layer, the weights of the weight matrix are distributed across the HP-PIM and LP-PIM modules according to a specific ratio, as shown in Figure 3, similar to the example in the convolution layer. Since the computation for each output node can be performed independently according to (2), the weight distribution across the PIM modules for the matrix–vector multiplication should ensure that the weights required to compute a single output node are contained within a single PIM module. In other words, for the column vector *X*, the rows of *W* must be stored in each PIM module to allow for parallel computation while minimizing data movement overhead.

The proposed heterogeneous PIM architecture, as demonstrated in the previous examples, can achieve optimal performance if the weights are appropriately allocated based on the characteristics of the computations within the application during the development process. Additionally, since the ratio of weights stored in the HP-PIM and LP-PIM modules reflects the proportion of computations each PIM module will handle, this allows for the adjustment of the balance between energy consumption and performance in the heterogeneous PIM. In the following section, we introduce a data placement strategy and discuss methods to optimize the energy consumption of the heterogeneous PIM during the application’s runtime.

## 3. Optimal Data Placement Strategy for the Proposed Heterogeneous PIM

The performance of the proposed heterogeneous PIM for target AI applications is closely related to the placement of the weight data. The overall computation results for each neural network layer are obtained by aggregating the results from multiple HP-PIM and LP-PIM modules within the heterogeneous PIM. In this process, even though the HP-PIM modules complete all assigned tasks quickly, there may be idle time as they wait for the slower LP-PIM modules to finish their computations. This idle time is directly tied to the performance of the PIM. To minimize it and ensure the PIM operates at its maximum performance, the workload allocation between the HP-PIM and LP-PIM modules must be carefully adjusted, allowing for the fastest possible inference results.

However, in real-time AI application processing, the application processes do not always demand the highest inference speed; in other words, they do not always require the PIM to operate at its maximum performance. When the inference frequency of the application is low, it is possible to satisfy the required latency without having the PIM operate at its highest throughput. In this case, more weights can be allocated to the energy-efficient LP-PIM modules to improve the overall energy efficiency of the processor. Leveraging this, we propose a weight placement strategy that periodically optimizes energy efficiency by adjusting the distribution of weights between the HP-PIM and LP-PIM modules during the application runtime.

The proposed weight placement strategy consists of two algorithms: one that determines the weights to be stored in the HP-PIM and LP-PIM modules during a given time period, and another that predicts the inference frequency of the next period in order to adjust the weight allocation ratio for the subsequent period. First, to explain the former in detail, the number of inferences $n_{task}$ performed during a given time period Δt, which is the interval during which a specific weight placement is maintained, is categorized into *N* levels based on the magnitude. The highest level, level *N*, corresponds to the maximum number of inferences $n_{task\_max}$ that the baseline processor with only HP-PIM modules (cf. Figure 1) can perform during Δt at its fastest operating speed. The remaining levels are then associated with the corresponding number of inferences, based on *N*, as follows:

(3) $$n_{task}\left(Level\;i\right)=\frac{n_{task\_max}}{N}*i\;\left(\mathrm{for}\;i=1,2,\cdots ,N\right).$$

**Figure 4:**
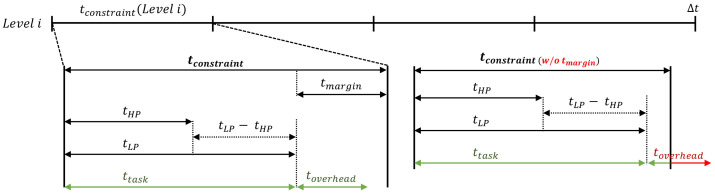
Relationship between time parameters in the proposed weight placement strategy.

To maintain a consistent inference latency for each inference frequency categorized by level, a time constraint $t_{constraint}$ must be set for the time in which each inference should be completed. Figure 4 illustrates the relationships between the time parameters in the proposed method. In the figure, $t_{HP}$ and $t_{LP}$ represent the time required for the HP-PIM and LP-PIM modules, respectively, to process all assigned computations, while $t_{task}$ refers to the time it takes to complete one inference across all PIM modules. As shown in the figure, since $t_{task}$ is directly affected by the computation time of the slower LP-PIM modules, $t_{constraint}$ can be determined based on the total computation time of the MAC operations performed by the LP-PIM modules. In other words, this defines how many weight data should be allocated to the LP-PIM modules to perform computations for each level. When defining $n_{weight\_LP}$ as the maximum number of weight data that can be stored in the LP-PIM modules under the given time constraint, $t_{constraint}$, $n_{weight\_LP}$ can be expressed as follows:

(4) $$t_{constraint}\left(Level\;i\right)=\frac{\Delta t}{n_{task}\left(Level\;i\right)}\;\left(\mathrm{for}\;i=1,2,\cdots ,N\right),$$

(5) $$n_{weight\_LP}\left(Level\;i\right)=\frac{t_{constraint}\left(Level\;i\right)}{t_{MAC\_LP}}\;\left(\mathrm{for}\;i=1,2,\cdots ,N\right),$$

where $t_{MAC\_LP}$ is the time required for a single MAC operation. Once $n_{weight\_LP}$ is determined for each level, the number of weight data stored in the HP-PIM modules, $n_{weight\_HP}$, is then determined as the remainder of the total weight data after subtracting $n_{weight\_LP}$. Based on these values, $n_{weight\_HP}$ and $n_{weight\_LP}$, the total weight data are evenly distributed across the multiple HP-PIM and LP-PIM modules.

Along with $n_{weight\_LP}$ for each level, the maximum number of weights that can be stored in the HP-PIM modules under the time constraint, denoted as $n_{weight\_HP}$, is stored in a lookup table and used for runtime data placement during the execution of the application. To derive the pre-calculated values of $n_{weight\_LP}$ and $n_{weight\_HP}$ to be stored in the table, we introduced an initialization phase. This phase involves storing all weights evenly across the HP-PIM modules and running a few inference tasks using test data before the application is fully deployed, while measuring the execution time.

However, relying solely on the table information filled during this initialization phase may be insufficient to address the additional time and energy overhead caused by weight placement operations, which repeat every Δt during runtime. These overheads cannot be captured during initialization because they vary depending on the level applied in the previous Δt. The potential problem that can arise if these overheads are not accounted for is depicted on the right-hand side of Figure 4. In this figure, it can be observed that the inference latency fails to meet the time constraint $t_{constraint}$ due to the overhead time $t_{overhead}$. Specifically, if the difference between the actual inference time $t_{task}$ and the time constraint $t_{constraint}$, denoted as $t_{margin}$, is smaller than $t_{overhead}$, the application’s required inference latency may be delayed.

To mitigate this issue, we introduced a turbo mode to the proposed PIM. This turbo mode defines a new level (N+1), with the fastest possible weight placement, and the corresponding $n_{weight\_HP}$ and $n_{weight\_LP}$ are also determined during the initialization phase. The turbo mode ensures that the inference time reduction exceeds the worst-case overhead difference of $t_{overhead}-t_{margin}$. Although the turbo mode could be further refined by introducing multiple levels for more granular control, this would increase design complexity, so we implemented only a single level in this work.

Next, we developed an algorithm to predict the inference frequency of the application for the next weight placement during the period Δt, in which the current weight placement is maintained. Various prediction methods, ranging from statistical techniques to machine learning approaches, can be applied. However, to ensure that the algorithm can be executed on edge devices and minimize overhead when integrated into existing applications, we adopted the lightweight and low-complexity simple exponential smoothing (SES) method. By using SES, which applies exponential weighting, the influence of the level applied in the previous Δt gradually diminishes, while more weight is assigned to the most recent Δt, allowing the inference frequency to be determined. This can be expressed by the following recursive formula:

(6) $$Level\_pred_{t_{0}+\Delta t}=\alpha *Level\_real_{t_{0}}+\left(1-\alpha \right)*Level\_pred_{t_{0}}\;\left(\mathrm{for}\;0\leq \alpha \leq 1\right),$$

where $Level\_pred_{t_{0}}$ and $Level\_pred_{t_{0}+\Delta t}$ represent the predicted levels at time $t_{0}-\Delta t$ and $t_{0}$, respectively, and $Level\_real_{t_{0}}$ refers to the actual inference frequency level during the previous Δt at time $t_{0}$. Additionally, α is the smoothing constant, and the closer this value is to 1, the more weight is placed on the most recent Δt level during prediction. We implemented an algorithm that maintains a table of the last 10 actual inference frequency levels that occurred over the previous 10Δt, updating it every Δt. The initial placement corresponds to the weight placement for level *N* and, until the level table is fully populated, predictions are made using only the actual level data gathered so far.

**Figure 5:**
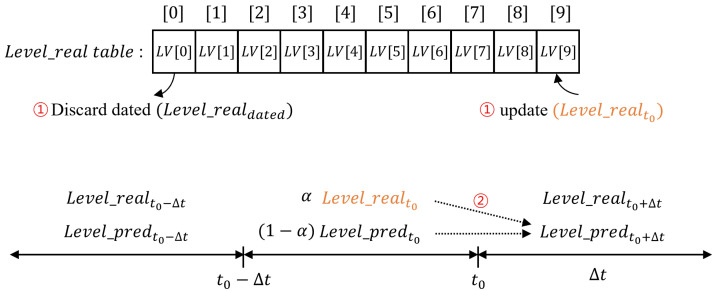
Prediction of inference occurrence level using the SES method.

Figure 5 shows the process through which the inference frequency level for the next Δt is predicted and the table is updated. First, after the weight placement is performed, the contents of the actual inference frequency level table are updated. Then, by iterating through elements 0 to 9 in the table and applying (6), the next inference frequency level for the upcoming Δt is predicted based on the accumulated data from the previous 10 actual inference frequency levels. However, there may be cases where the predicted frequency level for the next Δt is incorrect. If $Level_{pred}>Level_{real}$, even if the prediction fails, as long as $Level_{pred}<N$, the system can still achieve a certain degree of energy saving in the heterogeneous PIM, although it will not be optimal. On the other hand, if $Level_{pred}<Level_{real}$, the inference latency requirement may not be met. In such cases, the next weight placement will skip level prediction and immediately apply the weight placement corresponding to level (N+1) (turbo mode operation) to quickly handle the remaining inference requests.

## 4. Experimental Work

### 4.1. Experimental Setup

**Figure 6:**
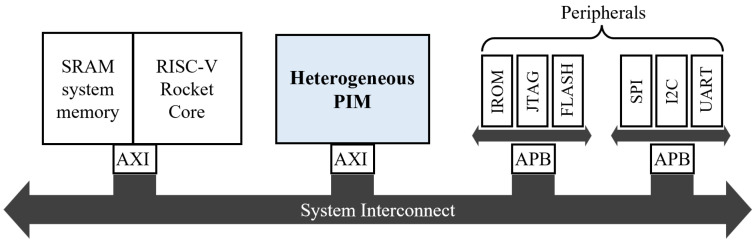
Architecture of the prototyped processor with the proposed heterogeneous PIM.

To validate the heterogeneous PIM architecture and evaluate the effectiveness of the data placement strategy in various dynamic scenarios of applications running on this architecture, we implemented both the baseline PIM and the heterogeneous PIM from Figure 1 at the RTL level using Verilog HDL. In this work, the baseline PIM consists of four HP-PIM modules, while the heterogeneous PIM is composed of four HP-PIM modules and four LP-PIM modules. Additionally, each PIM module contains 128 kB of MRAM and 2 kB of SRAM. Subsequently, we developed two processors using the RISC-V eXpress framework [26]: one with the baseline PIM and the other with the proposed heterogeneous PIM. Figure 6 shows the architecture of the processor equipped with the proposed PIM. As depicted, the developed processor uses a single core based on the RISC-V Rocket [27] core. The core and PIM are connected using the AXI protocol, which is well suited for high bandwidth and low latency, through the lightweight system interconnect known as μNoC [28,29].

To perform FPGA prototyping for the processor shown in Figure 6, and to verify its functionality and measure application execution speed, we first modeled the behavior and latency of the MRAM and SRAM at the RTL level. Table 1 and Table 2 present the read/write latency and dynamic/static power of HP-PIM and LP-PIM, obtained using simulation tools for MRAM and SRAM at 45 nm technology, under different operating voltages. The operating voltages were set to 1.2 V for HP-PIM and 0.8 V for LP-PIM, with the LP-PIM voltage specifically based on the latest specifications of fabricated MRAM chips [30,31]. For MRAM and SRAM, we used NVSim [32] and CACTI 7.0 [33], respectively. Other simulation parameters followed the default high-performance (HP) target process for HP-PIM and the low-operating-power (LOP) target process for LP-PIM, as provided by the software. For areas of the RTL design excluding memory, we synthesized the design using Synopsys Design Compiler [34] with the 45 nm Nangate PDK library [35], and the power consumption of the PE was derived from this synthesis. The computational latency of the PE was obtained by extracting the number of cycles from an RTL simulation of the designed heterogeneous PIM.

**Figure 7:**
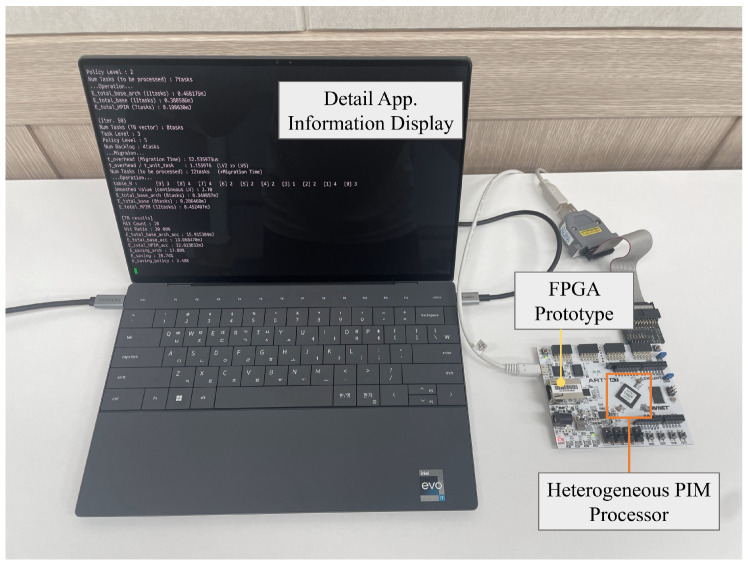
A demonstration of running a testbench on the FPGA prototype of the processor equipped with the heterogeneous PIM.

The FPGA prototyping of the processor was performed using the Arty-A7 FPGA board [36]. The prototype processor operates at a clock frequency of 50 MHz on the FPGA, with a clock period of 20 ns. However, as shown in Table 2, the read and write latency of SRAM in the HP-PIM is approximately 0.5 ns. To account for this discrepancy and ensure accurate performance evaluation of the proposed PIM, the latency values from the simulation results were scaled by a factor of 40 to match the clock frequency of the FPGA prototype. Figure 7 shows the FPGA prototyping process and the measurement of the proposed PIM’s performance using a testbench, while Table 3 reports the resource consumption results of the FPGA prototyping. Since only the timing modeling was applied to both the HP-PIM and LP-PIM modules in the proposed PIM FPGA prototype, the FPGA resource consumption for all PIM modules is identical.

### 4.2. Experimental Results

To verify whether the proposed heterogeneous PIM can dynamically respond to varying computational workloads and achieve energy savings through the proposed technology, we developed a testbench application and conducted experiments on the prototype processor under various inference demand scenarios. The testbench application, designed for an equal comparison between the baseline PIM and the proposed PIM, focuses solely on the MAC operations, which are key parallel components in neural networks. The application performs MAC operations on 1000 weight data and input data, treating this as a single task unit. The parameters were set to N=4, Δt=1 ms, and α=0.35, and the experiments were conducted over 50Δt, with various inference demand scenarios as inputs. The evaluation includes a comparison of energy consumption between the baseline PIM and the proposed PIM, as well as the energy-saving effect when the data placement strategy is applied in the proposed PIM. In the case where the data placement strategy is not applied, it means that the data storage configuration always corresponds to level *N* at every Δt.

**Figure 8:**
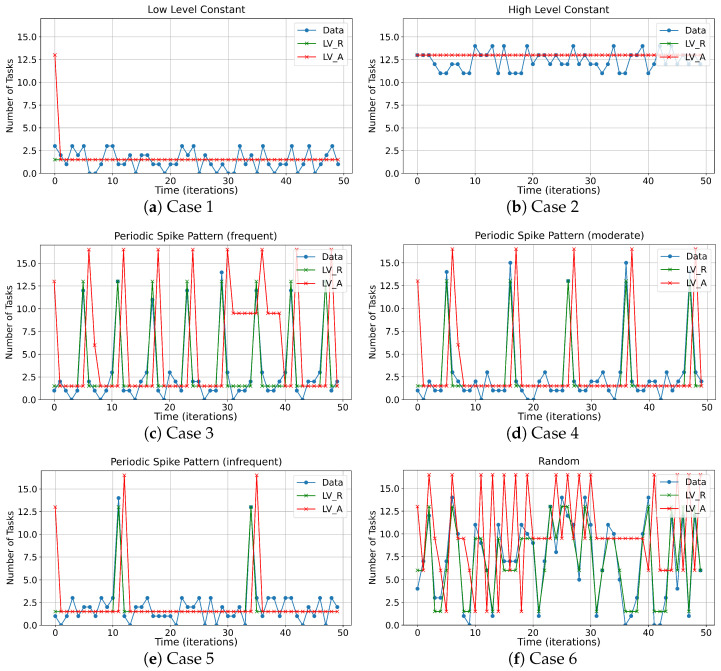
Measured results of data placement from the testbench application. The input pattern for each case is described at the top of the plot. The blue line indicates the number of tasks, while the green line shows Level_real. The red line represents Level_pred.

Figure 8 presents the plot of various inference demand scenarios used in the experiment and the corresponding results obtained from the developed testbench. The inference demand scenarios consist of input patterns such as low-level constant (case 1), high-level constant (case 2), frequent/moderate/infrequent periodic spike patterns (cases 3, 4, and 5, respectively), and a random pattern (case 6). The periodic spike pattern, in particular, represents a realistic scenario where computational load periodically surges, commonly observed in applications such as machine learning and image processing. In each case, the blue line indicates the number of tasks, while the green line shows the corresponding inference level, i.e., $Level\_real_{t_{0}}$, which is the actual level required at time $t_{0}$. The red line represents the level actually applied to the heterogeneous PIM based on the proposed data placement strategy, i.e., $Level\_pred_{t_{0}}$, which was predicted at $t_{0}-\Delta t$ and applied at $t_{0}$.

Table 4 reports the execution times of the HP-PIM and LP-PIM modules during the testbench application execution for each Δt in the proposed heterogeneous PIM architecture. At the highest level (level 5), both HP-PIM and LP-PIM record the same execution times, while at lower levels, fewer operations are allocated to HP-PIM, reducing its execution time, and more operations are assigned to LP-PIM, increasing its execution time. This workload distribution between HP-PIM and LP-PIM, achieved through the data placement algorithm, ensures that the execution time of LP-PIM does not exceed the $t_{constraint}$ for any level. Specifically, at level 1, all operations are assigned to LP-PIM, with an execution time of 159.88 μs, comfortably meeting the $t_{constraint}$ of 300 μs. The energy savings in the proposed architecture are primarily realized in lower levels through the dynamic power savings of HP-PIM.

Table 5 then reports the energy consumption of the baseline and proposed PIM for each testbench application in Figure 8 over 50 ms. In case 1, which consumed the minimum energy, the proposed PIM processor achieved 11.46% energy savings without the proposed data placement strategy and 29.51% energy savings with the strategy, compared to the baseline PIM processor. In the constant pattern case of case 2, the proposed PIM processor still achieved 18.96% energy savings over the baseline PIM processor. In this case, since the inference load remains at level *N* due to the testbench scenario, the use of the data placement strategy does not affect energy consumption. Notably, despite the proposed PIM processor incorporating four additional LP-PIM modules compared to the baseline, the LP-PIM modules significantly reduced the dynamic power consumption of the HP-PIM, leading to energy savings. In the periodic spike patterns of cases 3–5, the proposed PIM processor with the data placement strategy achieved energy savings of 21.07%, 23.82%, and 29.54%, respectively. Additionally, we observed that in these cases, when the task frequency in the application fluctuated too frequently, the increased use of level (N+1) data placement to meet the required computational latency reduced the degree of energy savings. Finally, in case 6, with the random pattern, the proposed PIM processor with the data placement strategy achieved 17.45% energy savings. However, we observed that the difference between applying the data placement strategy and not applying it was minimal. This is because the SES method we exploited is a statistical technique that predicts future patterns based on past data. Nonetheless, the introduction of the heterogeneous PIM architecture still demonstrated significant double-digit energy savings, even in scenarios with irregular computation load variations.

## 5. Conclusions

In this paper, we have introduced a novel PIM architecture designed to adapt in real time to the dynamic computational workloads of edge applications. To achieve optimal energy efficiency, we have proposed an operational strategy tailored to this architecture. The core of our design features two distinct types of PIM modules: low-power PIM (LP-PIM) modules and high-performance PIM (HP-PIM) modules. This heterogeneous configuration enables the architecture to flexibly handle varying workloads in real time, offering a high degree of adaptability to fluctuating computational demands. Additionally, we have developed a data placement strategy aimed at maximizing the energy efficiency of the proposed heterogeneous PIM architecture. This strategy optimally distributes data between the HP-PIM and LP-PIM modules based on an algorithm that predicts workload changes during application execution, ensuring efficient use of resources. To validate the effectiveness of our proposed solution, we have implemented the PIM architecture and developed an embedded RISC-V processor incorporating the proposed PIM. Through FPGA prototyping, we have successfully verified the functionality of the processor. Performance evaluations conducted under a range of computational scenarios have shown that the proposed technology achieves up to 29.54% energy savings, demonstrating its significant potential for energy-efficient AI applications on edge devices.

## Figures and Tables

**Table 1:** Power consumption comparison between the HP-PIM and LP-PIM modules.

Power (mW)	MRAM (128 kB)		SRAM (2 kB)		PE	
	**Dynamic (Read/Write)**	**Static**	**Dynamic (Read/Write)**	**Static**	**Dynamic**	**Static**
HP-PIM ($V_{dd}=1.2$ V)	36.45/65.8	3.68	34.7/41.3	3.94	0.9	0.48
LP-PIM ($V_{dd}=0.8$ V)	17.01/45.36	0.016	9.21/10.45	0.051	0.51	0.25

**Table 2:** Latency comparison between the HP-PIM and LP-PIM modules.

Latency (ns)	MRAM		SRAM		PE
	**Read**	**Write**	**Read**	**Write**	
HP-PIM ($V_{dd}=1.2$ V)	2.27	6.17	0.46	0.46	4.72
LP-PIM ($V_{dd}=0.8$ V)	3.44	7.16	0.89	0.89	7.42

**Table 3:** FPGA resource consumption results.

IP		LUTs	FFs
RISC-V Rocket		11,375	5762
Peripherals		5318	8607
System Interconnect		4624	6070
Heterogeneous PIM (PIM Module ×8)		34,757	13,832
	• PIM Module	3809	1462
	• PIM Controller	1767	871

**Table 4:** Execution times of HP-PIM and LP-PIM and $t_{constraint}$ during each Δt for each level.

Execution Time (μs)	Level 1	Level 2	Level 3	Level 4	Level 5
$t_{constraint}$	300.00	128.57	90.00	64.29	-
HP-PIM	0	12.43	27.91	38.05	45.41
LP-PIM	159.88	128.54	89.53	63.95	45.41

**Table 5:** Energy consumption results for cases 1 to 6.

Total Energy Consumption (mJ)	Case 1	Case 2	Case 3	Case 4	Case 5	Case 6
Baseline PIM	4.53	26.22	8.53	7.5	6.39	14.9
Hetero-PIM (w/o data placement)	4.01	21.25	7.19	6.37	5.49	12.41
Hetero-PIM (with data placement)	3.19	21.25	6.73	5.71	4.5	12.3

## Data Availability

The original contributions presented in the study are included in the article, further inquiries can be directed to the corresponding author.
